# An update of clinical value of circulating tumor DNA in esophageal cancer: a systematic review and meta-analysis

**DOI:** 10.1186/s12885-024-11879-6

**Published:** 2024-01-24

**Authors:** Yaozhong Zhang, Huazhen Du, Na Wang, Lei Wang, Yajie Huang

**Affiliations:** 1 Department of Infectious diseases, the Fourth Hospital of Hebei Medical University, Shijiazhuang, China; 2 Department of Emergency, the Fourth Hospital of Hebei Medical University, Shijiazhuang, China; 3 Department of Molecular Biology, the Fourth Hospital of Hebei Medical University, Shijiazhuang, China; 4 Department of Thoracic Surgery, the Fourth Hospital of Hebei Medical University, Shijiazhuang, China; 5 Department of Medical oncology, the Fourth Hospital of Hebei Medical University, Shijiazhuang, China

**Keywords:** Circulating tumor DNA (ctDNA), Esophageal cancer (EC), Droplet digital polymerase chain reaction (ddPCR), Meta-analysis, Next-generation sequencing (NGS)

## Abstract

**Background:**

Esophageal cancer (EC) is a deadly disease with limited therapeutic options. Although circulating tumor DNA (ctDNA) could be a promising tool in this regard, the availiable evidence is limited. We performed a systematic review and meta-analysis to summarize the clinical applicability of the next-generation sequencing (NGS) and droplet digital polymerase chain reaction (ddPCR) technology on the ctDNA detection of the EC and listed the current challenges.

**Methods:**

We systematically searched MEDLINE (via PubMed), Embase (via OVID), ISI Web of Science database and Cochrane Library from January, 2000 to April, 2023. Progression-free survival (PFS) and overall survival (OS) were set as primary outcome endpoints. Pathologic response was evaluated by tumor regression grade (TRG), according to the eighth edition of the American Joint Committee on Cancer (AJCC). Major pathologic regression (MPR) was defined as TRG 1 and 2. The MPR was set as secondary endpoint. Hazard rate (HR) and associated 95% CI were used as the effect indicators the association between ctDNA and prognosis of EC. MPR rates were also calculated. Fixed-effect model (Inverse Variance) or random-effect model (Mantel-Haenszel method) was performed depending on the statistically heterogeneity.

**Results:**

Twenty-two studies, containing 1144 patients with EC, were included in this meta-analysis. The results showed that OS (HR = 3.87; 95% CI, 2.86–5.23) and PFS (HR = 4.28; 95% CI, 3.34–5.48) were shorter in ctDNA-positive patients. In the neoadjuvant therapy, the sensitivity analysis showed the clarified HR of ctDNA-positive was 1.13(95% CI, 1.01–1.28). We also found that TP53, NOTCH1, CCND1 and CNKN2A are the most frequent mutation genes.

**Conclusions:**

Positive ctDNA is associated with poor prognosis, which demonstrated clinical value of ctDNA. Longitudinal ctDNA monitoring showed potential prognostic value in the neoadjuvant therapy. In an era of precision medicine, ctDNA could be a promising tool to individualize treatment planning and to improve outcomes in EC.

**PROSPERO registration number:**

CRD42023412465.

**Supplementary Information:**

The online version contains supplementary material available at 10.1186/s12885-024-11879-6.

## Introduction

Esophageal cancer (EC) ranks the eighth most commonly diagnosed cancer and the sixth most common cause of cancer-related mortality worldwide [1]. According to the latest data of China National Cancer Center, esophageal cancer ranked the sixth and the mortality ranked the fourth [2]. There are two main histological subtypes of EC, esophageal adenocarcinoma (EAC) and esophageal squamous cell carcinoma (ESCC). The ESCC accounts for about 90% of EC patients; while, the prevalence rate of EAC have paralleled increases with the change of diet and rising of obesity rates [3]. EC carried a poor prognosis: the five-year survival rate of ESCC was only 35–45% [4] and the five-year survival rate of EAC was even lower [3]. At present, the neoadjuvant therapy is widely applied to improve long-term survival rate in clinical trials [5, 6]. The neoadjuvant therapy mainly included neoadjuvant chemotherapy(nCT) and neoadjuvant chemoradiotherapy(nCRT). Currently, one of the challenges of the EC anticancer therapy is preventing patients from undergoing unnecessary esophagectomy. In the CROSS trial, 30% of patients received neoadjuvant treatment with distant metastases within two years after surgery [7]. The routinely diagnostic and staging investigations of EC patients include computed tomography scans (CT scan), positron emission tomography (PET) scans, endoscopic ultrasound (EUS) and endobronchial ultrasound (EBUS). However, these investigations cannot reflect pathological characteristics or are too invasive to be used repeatedly [8–10]. Hence, a reliable biomarker would be meaningful for the clinical neoadjuvant therapy, which can be tested and tracked noninvasively to forecast the result of tumor treatment.

More recently, some studies have demonstrated the clinical value of circulating tumor DNA (ctDNA) for EC [11, 12]. The ctDNA is defined as cell free DNA (cfDNA) released by necrotic and apoptotic cancer cells into the blood; and its diagnostic, therapeutic, and prognostic value have been widely studied. There are two main technological strategies: next-generation sequencing (NGS) and droplet digital polymerase chain reaction (ddPCR) [13, 14]. Several studies concentrated on the clinical value on the NGS technology application in the ctDNA detection in EC patients [15, 16]. And, the NGS technology improves mutation detection rate in ctDNA samples with lower cost and higher efficiency [17]. The ddPCR can be used to directly quantify and clonally amplify DNA, which is a refinement of the conventional polymerase chain reaction methods [18]. Advances in genome sequencing, including NGS and ddPCR, have made detection and analysis of ctDNA more feasible. However, advantages and shortcomings of two technologies are inconclusive.

One published meta-analysis demonstrated that the ctDNA is a potential biomarker for diagnosis and monitoring of EC patients, with a moderate sensitivity and high specificity. However, no such analysis compared the advantages and shortcomings between the two technologies or evalued the clinical significance of ctDNA in two histological subtypes in the published meta-analysis [19]. Therefore, it is meaningful to comprehensively assess the clinical value of ctDNA in EC. This systematic review and meta-analysis firstly summarized the clinical applicability of NGS and ddPCR technology on ctDNA detection of the EC and listed its current challenges, and evaluated the predictive efficiency of neoadjuvant therapy response.

## Methods

This study was performed in line with the Preferred Reporting Items for Systematic Reviews and Meta-analyses (PRISMA) reporting guidelines [20, 21] (checklists were presented in the Supplement). All data were extracted from previous ethically approved studies; therefore, patient consent and ethical approval were not required [22]. The protocol of this study had been registered in the International Prospective Register of Systematic Reviews (PROSPERO), under the registration number of CRD42023412465.

### Search strategy

MEDLINE (via PubMed), Embase (via OVID), ISI Web of Science database and Cochrane Library were browsed for eligible studies from January, 2000 to April, 2023. The analysis only searched English databases. The following search terms or keywords were used: Circulating Tumor DNA (MeSH) OR Cell Free Tumor DNA OR ctDNA AND esophageal carcinoma (MeSH) OR esophageal cancer OR oesophageal cancer AND High Throughput Nucleotide Sequencing (MeSH) OR Next Generation Sequencing OR Illumina Sequencing OR Deep Sequencing. The last search was conducted on April 27, 2023. All titles and abstracts were were screened and reviewed carefully. If no sufficient data in publications were extracted, the authors contacted the corresponding authors to get relevant data to analysis.

### Inclusion and exclusion criteria

Two authors (Y.Z. and Y.H.) independently retrieved the available literature to identify the eligible studies. The studies were chosen on the basis of the following criteria: (a) the study had a cohort or case–control design; (b) survival data was reported and could estimate a hazard ratio (HR) with 95% confidence interval (CI); (c) a ctDNA blood sample was measured; (d) the detection of ctDNA was based on NGS platform or ddPCR platform; and (e) the study only included patients with EC. We identified the esophagogastric junction adenocarcinoma as the EAC due to their similarity in clinical features and therapeutic strategies. On the contrary, studies beyond the inclusion criteria were excluded. Exclusion criteria were as the following criteria: (a) Insufficient data and unable to calculate data of interest; (d) multiple primary cancer;and (c) case report, comment, letter, review, and meta-analysis.

### Data extraction and quality assessment

Two reviewers (Y.Z. and H.D.) independently performed data extraction and quality assessment of all eligible studies. The following information was collected: first author, year of publication, region, characteristics of the study population (number, sex and age), sequencing platform, detection gene, ctDNA-positive rate, treatment therapy, follow-up period, survival data and its associated standard errors on prognostic outcomes. If the hazard ratio (HR) and their 95% CI were not directly provided in the original articles, the extracted survival information and the published risk table were used to reconstruct the survival curve for each included study using the method of David [23]. The extraction of information was repeated if two reviewers can’t achieve consensus. Pathologic response was evaluated by tumor regression grade (TRG), according to the eighth edition of the American Joint Committee on Cancer (AJCC). Major pathologic regression (MPR) was defined as TRG 1 and 2 [24].

The methodological quality was assessed by two reviewers (Y.Z. and H.D.) and the Newcastle-Ottawa Scale (NOS) was applied. The low quality study was defined as 0–3 scores, and 7–9 scores was defined as high quality [25]. Two reviewers resolved their discrepancies through discussion and consensus. An additional adjudicator (N.W.) would be invited into the discussion to made final decision if no agreement was reached after two reviewers discussed.

### Statistical analysis

Statistical analysis was performed utilizing the software R (version 4.2.3, R Foundation for Statistical Computing) via the meta package, in RStudio (version 4.0.4, RStudio). HR, RR and their associated 95% CI were used as the effect indicators for outcome data reported to summarize the prognostic significance of ctDNA in EC. All eligible studies were included in the analysis. Any HRs reported in the studies were used when available; otherwise, they were extrapolated using the available data. First, we extracted and calculated survival data such as overall survival (OS), progression-free survival (PFS), disease-free survival (DFS), or relapse-free survival (RFS). Heterogeneity across studies was measured with *Q* and *I*^*2*^ statistics [26]. Studies with an *I*^*2*^ statistics of 0%, 25%, 50% and 75% which are corresponding to the no, low, moderate, and high heterogeneity. The pooled HRs with 95% CI were calculated using a random-effect model [27], when there were moderate or high heterogeneity. If data was with no or low heterogeneity, fixed-effect model was applied. Sensitivity analysis, subgroup analysis and cumulative meta-analysis were all performed to explore the sources of heterogeneity. The potential publication bias was further validated by the Egger’s and Begg’s test [28]. All statistical analyses were two sides; and *P* value less than 0.05 was considered statistically significant.

## Results

### Study selection and characteristics

**Fig. 1:**
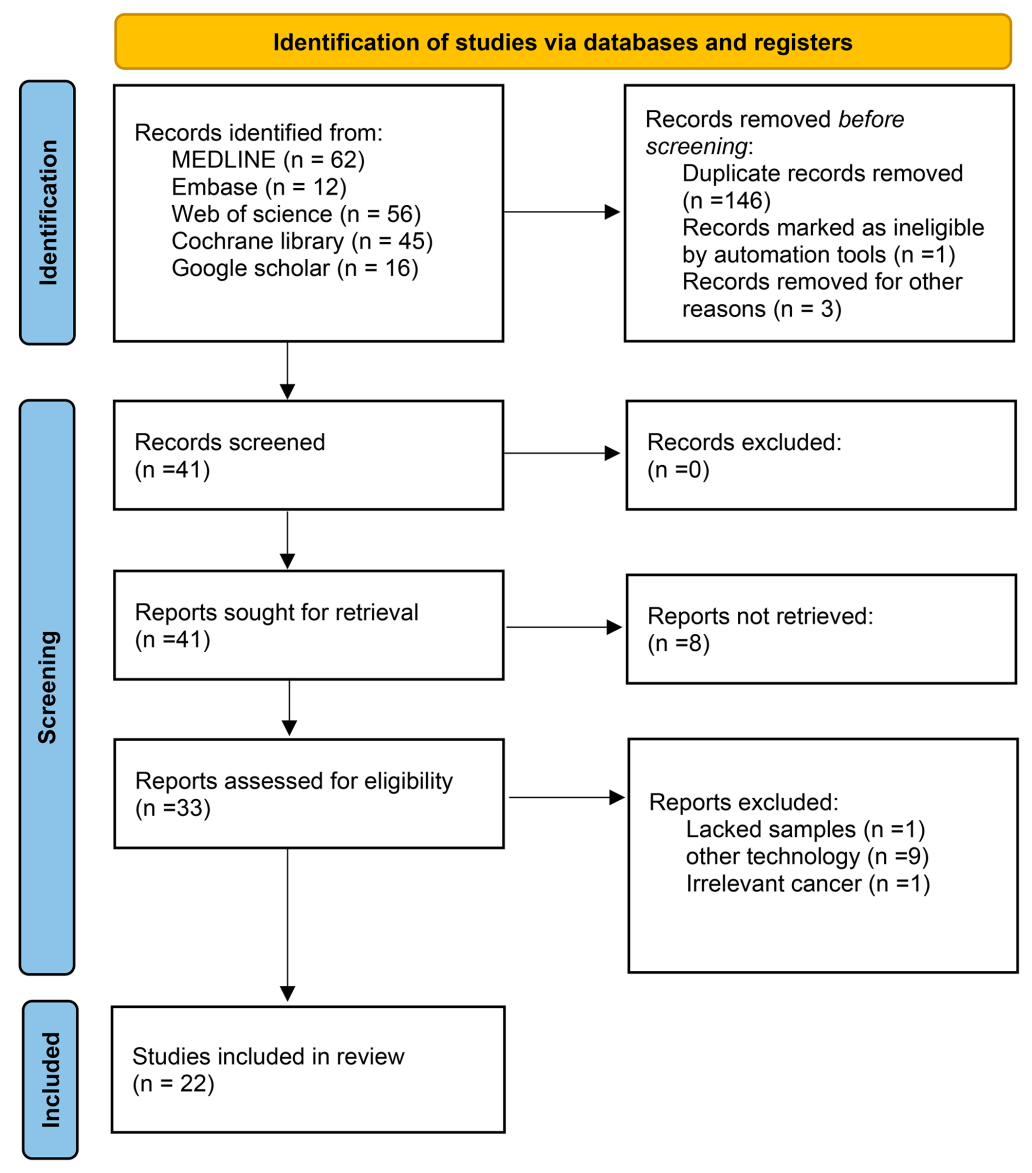
PRISMA flow diagram of study selection process

A total of 191 records were identified and included. All investigators finally agreed to incorporate twenty-two eligible studies [12, 16, 29–48] with 1144 patients in the meta-analysis. The PRISMA flow chart of this meta-analysis was shown in Fig. 1. Among the eligible studies, eleven studies [30, 31, 34–39, 41, 43, 46] were conducted on ESCC, and thirteen studies [12, 16, 29, 31–33, 37, 40, 42, 44, 45, 47, 48] addressed EAC. Seventeen studies [12, 16, 30–32, 34, 35, 37–43, 45, 47, 48] were based on the NGS; while the ddPCR technology was applied in five studies [29, 33, 36, 44, 46]. The characteristics of each study included in the current meta-analysis are reported in Table 1. Both two reviewers agreed on review of the extracted data. All studies were of moderate or high quality. The Newcastle-Ottawa scores are presented in the Supplement.

**Table 1:** Characteristics of included studies for the meta-analyses

Study	Year	Country	Method (panel)	endpoint	Patient		Treatment regimens
					Number	Male/Female	
Ococks E [29]	2021	UK	NextSeq550 Illumina (77 genes)	PFS/OS	97	83/14	nCT
Jia R [30]	2021	China	Illumina HiSeq3000 (180 genes)	PFS/OS	24	20/4	Radiation
Azad TD [31]	2020	US	CAPP-seq (607 genes)	PFS/OS	45	30/15	CRT Surgery
Davidson M [32]	2019	UK	Illumina HiSeq2500 (182 genes)	PFS/OS	30	26/4	chemotherapy
Openshaw MR [33]	2020	UK	ddPCR (6 genes)	PFS/OS	40	33/7	chemotherapy
Maron SB [12]	2019	US	Guardant360 test (73 genes)	OS	183	NG	Chemotherapy Surgery
Luo H [34]	2016	China	Illumina TruSight Cancer (94 genes)	PFS/OS	11	NG	Surgery
Liu T [35]	2021	China	Illumina HiSeq2500 (61 genes)	PFS/OS	55	49/6	chemotherapy
Hsieh CC [36]	2016	Japan	ddPCR (NG)	PFS/OS	81	NG	Chemotherapy Surgery CRT
van Velzen MJM [16]	2022	Netherland	Ion Torrent 2500/500 (NG)	PFS/OS	72	56/16	chemotherapy
Hofste LSM [37]	2022	Netherland	NextSeq500 Illumina (15 genes)	PFS/OS	78	60/18	nCRT
Wang X [38]	2022	China	Geneseeq (474 genes)	PFS/OS	40	34/6	Radiotherapy CRT
Zhang R [39]	2020	China	Illumina HiSeq X Ten (NG)	PFS/OS	22	14/8	nCT
Eyck BM [40]	2022	Netherland	Ion Torrent S5 GeneStudio Prime System (NG)	PFS/OS	31	NG	nCRT
Morimoto Y [41]	2023	Japan	NextSeq Illumina (77 genes)	PFS	13	10/3	nCT
Cabalag CS [42]	2022	Australia	MiSeq Illumina (48 genes)	PFS/OS	62	NG	nCRT
Yang D [43]	2022	China	NGS (1021 genes)	PFS	10	9/1	nCIT
Wallander K [44]	2023	Sweden	ddPCR (30 genes)	PFS/OS	10	6/4	CRT
Fujisawa R [46]	2021	Japan	ddPCR (30 genes)	PFS/OS	42	NG	nCT
Mehta R [48]	2023	US	Signatera MRD (NG)	OS	53	NG	NG
Lander EM [47]	2023	US	Signatera MRD (NG)	OS	34	NG	nCRT
van den Ende T [45]	2023	Netherland	NGS (23 genes)	PFS/OS	111	NG	nCRT

NG: Not Given; nCT: Neoadjuvant Chemotherapy; nCRT: Neoadjuvant Chemoradiotherapy; nCIT: Neoadjuvant chemoimmunotherapy; CRT: Chemoradiotherapy; NGS: next-generation sequencing; ddPCR: digital polymerase chain reaction

### Correlation between ctDNA and OS

**Fig. 2:**
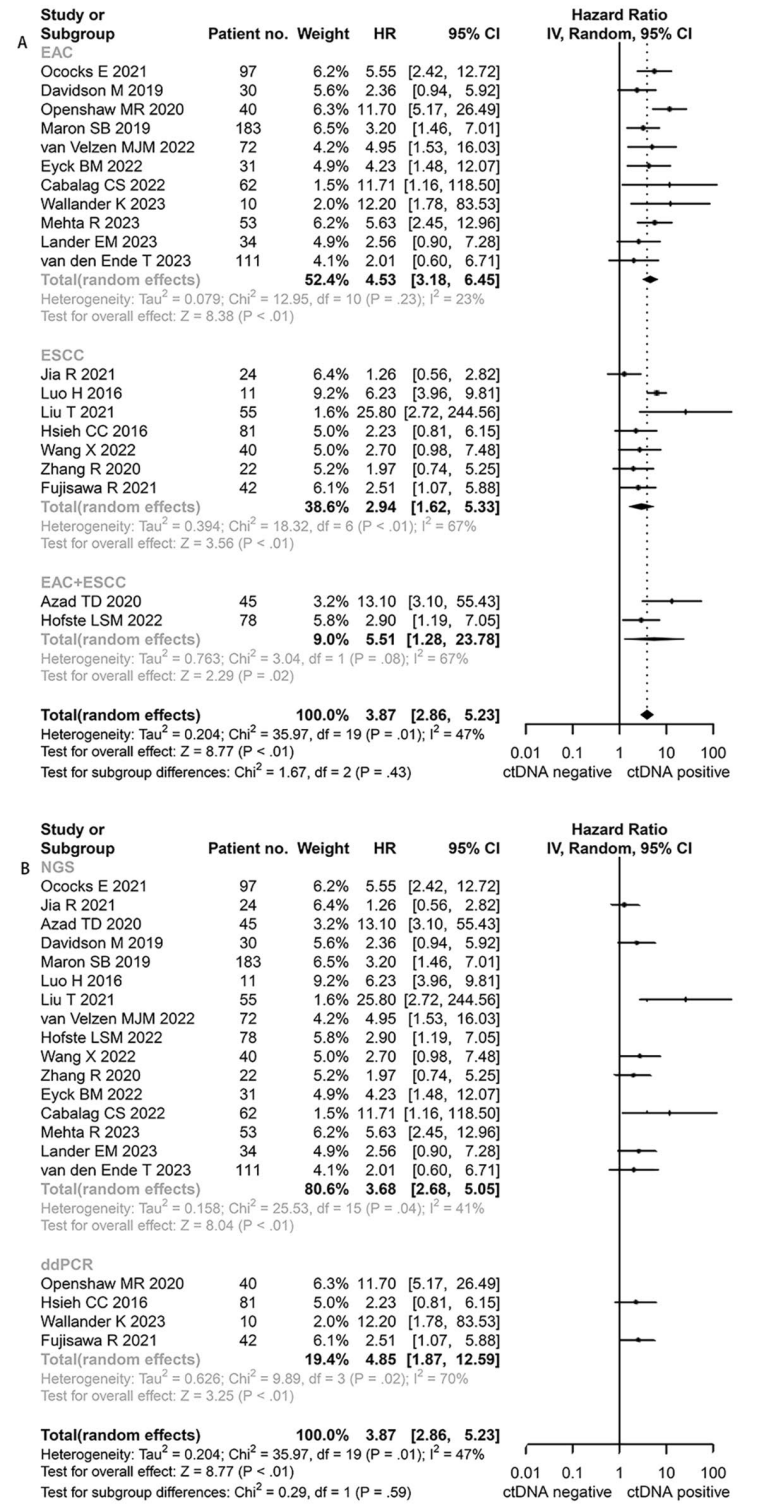
Hazard ratio (HR) for overall survival (OS) of the included studies. **A**: subgroup-analysis based on pathology, EAC: esophageal adenocarcinoma; ESCC: esophageal squamous cell carcinoma; **B**: subgroup-analysis based on method, NGS: next-generation sequencing (NGS); ddPCR: droplet digital polymerase chain reaction

Twenty studies were included in the OS meta-analysis [12, 16, 29–40, 42, 44–48]. The random-effect model was applied to calculated the pooled HR due to the moderate heterogeneity between studies (*I*^2^ = 47%, *P* = 0.43). A positive association was observed between the lower OS of the EC patients and the ctDNA-positive EC patients (HR = 3.87; 95% CI, 2.86–5.23, Fig. 2).

The results of subgroup analysis show the negative correlation between ctDNA-positive and OS in two histologic subtypes. Twenty studies were divided into three subgroups (EAC group, ESCC group and EAC plus ESCC group). The estimated HR was 4.53 (95% CI, 3.18–6.45, Fig. 2A) in the EAC subgroup, with lower heterogeneity (*I*^2^ = 23%, *P* = 0.23); while, the ESCC subgroup showed high heterogeneity (*I*^2^ = 67%, *P* < 0.01) and the HR was 2.94 (95% CI, 1.62–5.33, Fig. 2A). We also compared the two technology platforms. The estimated HR was 3.68 (95% CI, 2.68–5.05, Fig. 2B) in the NGS subgroup, with moderate heterogeneity (*I*^2^ = 41%, *P* = 0.04); while the ddPCR subgroup showed similar result (HR = 4.85, 95% CI 2.68–5.05, Fig. 2B) with high heterogeneity (*I*^2^ = 70%, *P* = 0.02). The sensitivity analysis and cumulative analysis showed that no single study markedly changed the primary outcome. Hence, the result was reliable and stable.

### Correlation between ctDNA and PFS

**Fig. 3:**
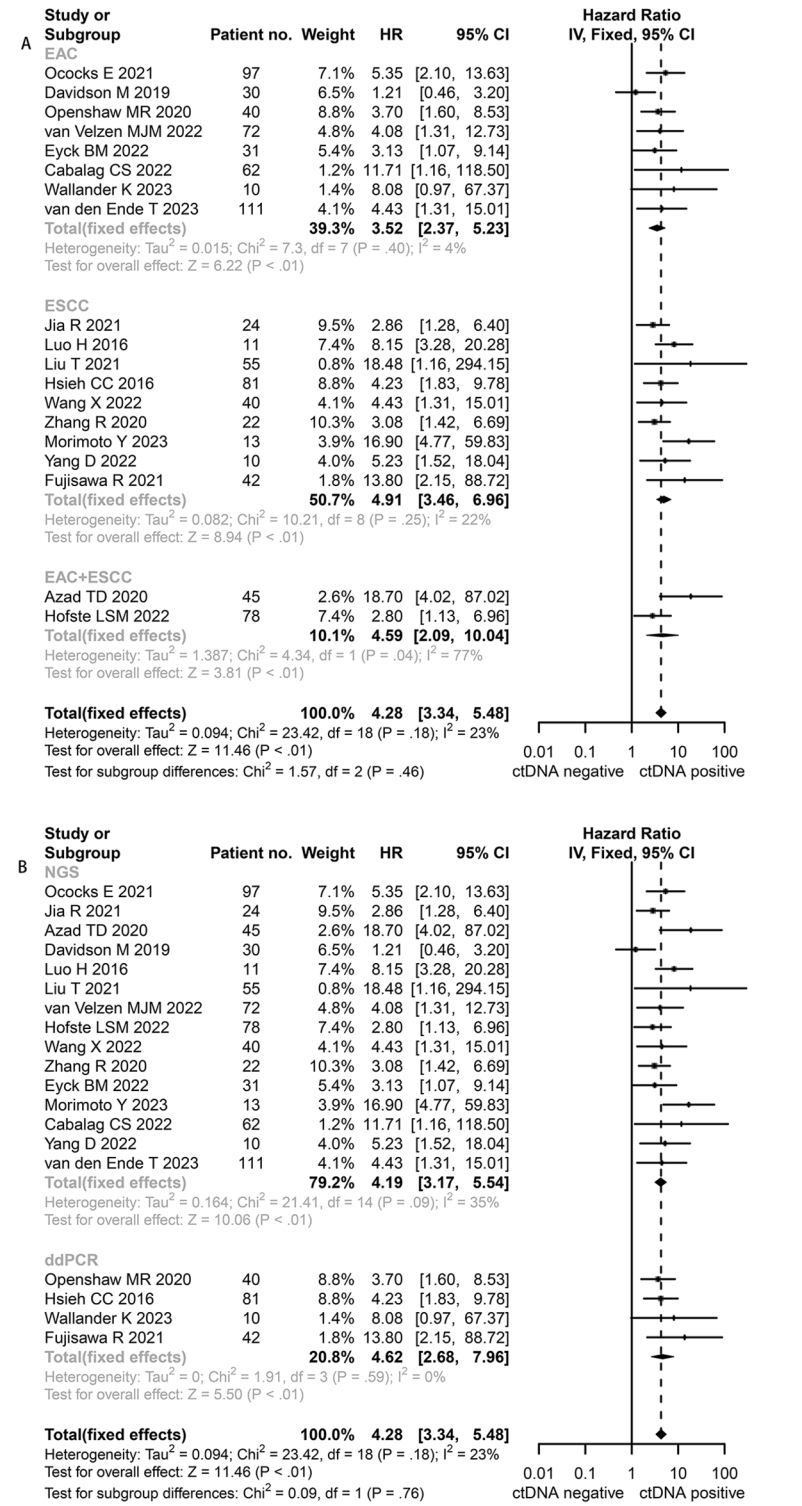
Hazard ratio (HR) for progression-free survival (PFS) of the included studies. **A**: subgroup-analysis based on pathology, EAC: esophageal adenocarcinoma; ESCC: esophageal squamous cell carcinoma; **B**: subgroup-analysis based on method, NGS: next-generation sequencing (NGS); ddPCR: droplet digital polymerase chain reaction

Nineteen studies reported on the PFS of the ctDNA [16, 29–46]. As the range of HR between these studies was 1.21 to 18.70, a significant association was observed between ctDNA-positivity in EC patients and the poorer PFS. Due to the low heterogeneity among the studies (*I*^2^ = 23%, *P* = 0.18), we applied the fixed-effect model to calculate. The pooled HR was 4.28 (95% CI, 3.34–5.48, Fig. 3).

In the subgroup analysis, the estimated HR of the EAC subgroup was 3.52 (95% CI, 2.37–5.23, Fig. 3A), with heterogeneity (*I*^2^ = 4%, *P* = 0.40); and, the ESCC subgroup showed high heterogeneity (*I*^2^ = 22%, *P* = 0) and the HR was 4.62 (95% CI, 2.68–7.96, Fig. 3A). The pooled results of the NGS platform and the ddPCR platform were 4.19 (95% CI, 3.17–5.54, Fig. 3B) and 4.62 (95% CI, 2.68–7.96, Fig. 3B), respectively. All subgroups were with low heterogeneity. The sensitivity analysis and cumulative analysis showed that the differences in the studies were not statistically significant.

### Clinical value for neoadjuvant therapy

**Fig. 4:**
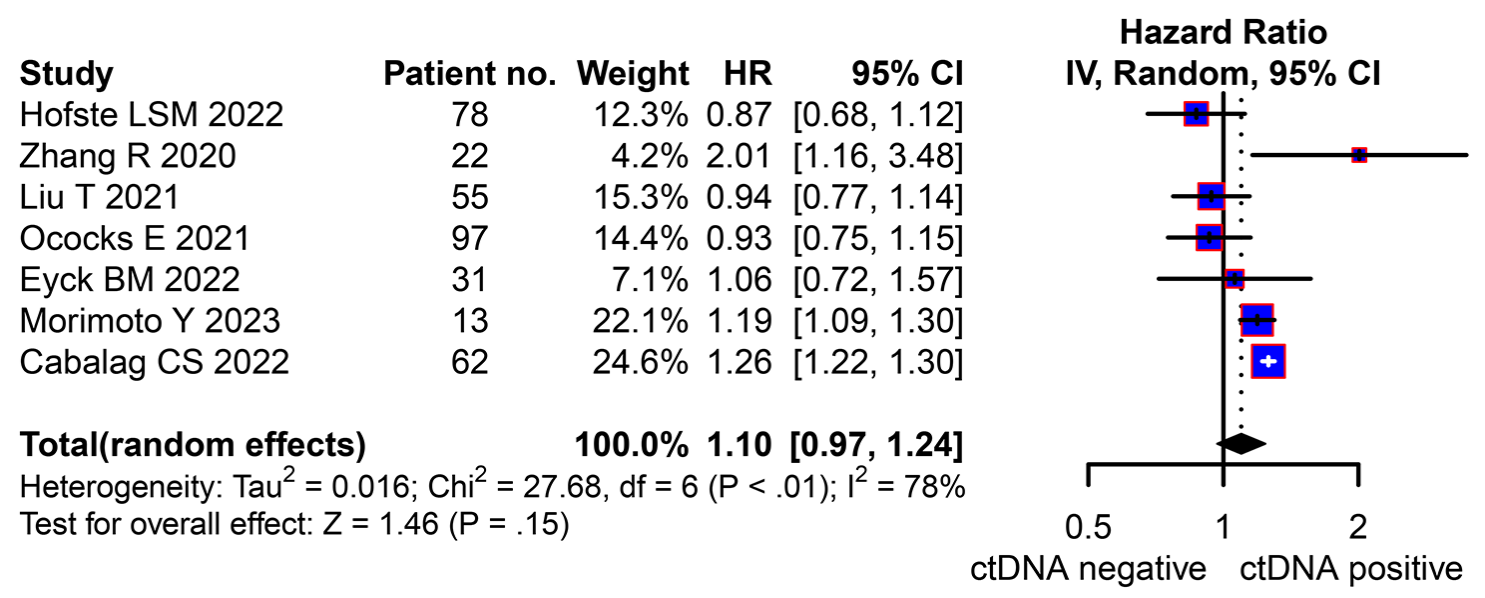
Hazard ratio (HR) for circulating tumor DNA (ctDNA) of neoadjuvant therapy

**Fig. 5:**
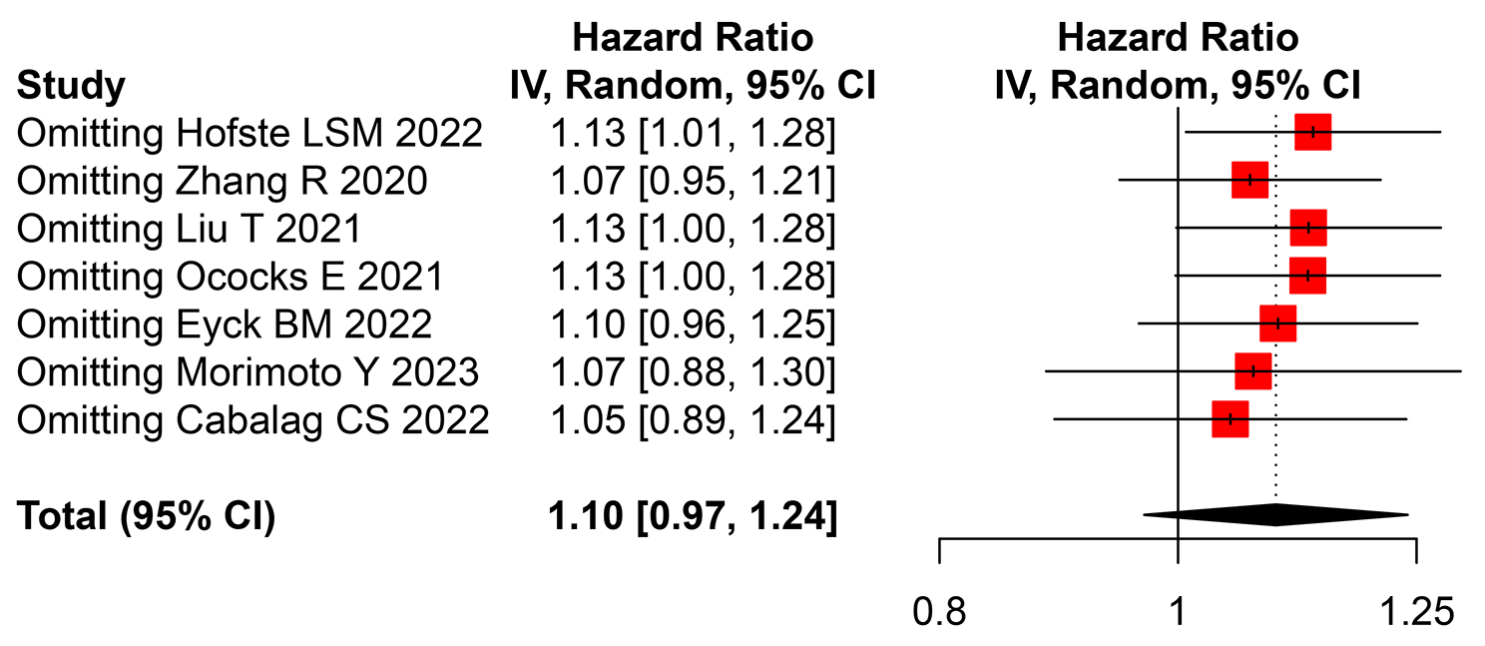
Sensitivity analysis of hazard ratio (HR) for circulating tumor DNA (ctDNA) of neoadjuvant therapy

**Fig. 6:**
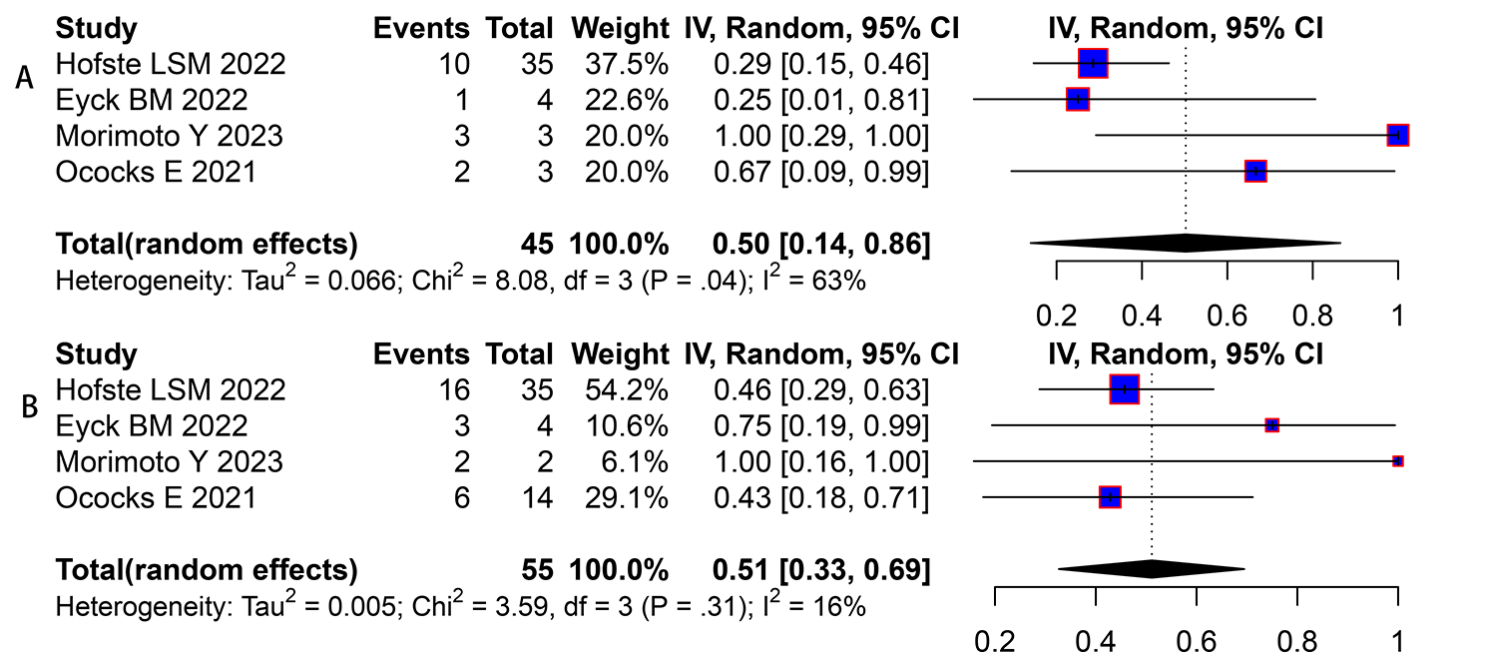
(**A**) The estimated major pathologic regression (MPR) rate of patients circulating tumor DNA (ctDNA) changed positive to negative; (**B**) The estimated MPR rate of patients ctDNA kept negative from beginning to end

There were seven studies provided preoperative data of the ctDNA about neoadjuvant therapy [29, 35, 37, 39–42]. The pooled HR of the preoperative ctDNA was 1.10 (95% CI, 0.97–1.24, Fig. 4) with high heterogeneity (*I*^2^ = 78%, *P* < 0.01). While, in the sensitivity analysis, the clarified HR was 1.13(95% CI, 1.01–1.28, Fig. 5) still with high heterogeneity (*I*^2^ = 75%, *P* < 0.01). Four eligible studies were included to analysis the clinical value of the longitudinal ctDNA for disease monitoring [29, 37, 40, 41]. The estimated MPR rate was 50% (95%CI:14-86%, *I*^2^ = 63%, Fig. 6A) of patients changed positive to negative; and 51% (95%CI:33-69%, *I*^2^ = 16%, Fig. 6B) for patients kept negative from beginning to end.

### Publication bias

The Newcastle-Ottawa Scale for quality appraisal of the included studies was presented in the Supplement. All studies were moderate or high quality, score ranged from 6 to 9. We used the trim and fill method and the conclusions were not changed [49]. It has to be noted that the assessment of publication bias is weak because of the small number of studies.

### Significantly mutated genes

With regard to the ctDNA detected studies [31, 50], it is important to identify the mutated genes rate, which is useful to design the gene panel. Based on the included twenty-two studies [12, 16, 29–48], TP53 was the most frequent detected mutation. However, most of the TP53 variations were either missense or non‑sense. Other frequently mutated genes included NOTCH1, CCND1 and CNKN2A (Table 2). Notably, the majority of the mutated genes with a high prevalence rate all were tumor suppressor genes.

**Table 2:** Significantly Mutated Genes

Study	Significantly Mutated Genes	Total
**ESCC**		
Jia R [30]	TP53(41.6%) NOTCH1(16.7%) CCND1(12.5%)	24
Luo H [34]	TP53(75%) NOTCH1(25%)	11
Liu T [35]	TP53(56.6%) PIK3CA(16.9%)	55
Hsieh CC [36]	NG	
Wang X [38]	TP53(85.7%) PRSS3(21.4%) CDKN2A(17.9%) ART(14.3%)	40
Zhang R [39]	NG	
Fujisawa R [46]	TP53(78.3%), NFE2L2 (12.1%) AJUBA(9.1%)	42
Morimoto Y [41]	TP53(85%) CDKN2A(9.1%) NFE2L2 (9.1%)	11
Yang D [43]	TP53(75%)	10
**EAC**		
Ococks E [29]	TP53(15%) APC(8%) KRAS(6%)	97
Davidson M [32]	NG	24
Openshaw MR [33]	TP53(84%) CCND1(24%) CCNE1(14%) VEGFA(14%)	40
Maron SB [12]	TP53(53%) HER2 (17%) EGFR (17%) KRAS (15%)	183
van Velzen MJM [16]	TP53(60%) KRAS(22%)	72
Eyck BM [40]	TP53(87%) CDKN2A(16.1%) KRAS (9.7%) SMAD4 (6.5%)	31
Cabalag CS [42]	TP53(80%)	62
Wallander K [44]	NG	10
van den Ende T [45]	NG	
Mehta R [48]	NG	
Lander EM [47]	NG	
**EAC + ESCC**		
Azad TD [31]	TP53(71.1%) ERBB2(11.1%) CDKN2A(6.3%)	45
Hofste LSM [37]	TP53(60%)	78

NG: Not Given; EAC: esophageal adenocarcinoma; ESCC: esophageal squamous cell carcinoma

## Discussion

Our study suggested that ctDNA-positive patients had poorer OS and DFS than ctDNA-negative patients in patients with EC, including EAC and ESCC. This meta-analysis also demonstrated that both the ddPCR platform and the NGS platform were effective in detection of ctDNA in EC patients. In addition, we also evaluated the clinical predicted value of ctDNA mutation profiles in patients with EC who received neoadjuvant therapy; and we found the pre-surgery ctDNA-positive were associated with the worse pathology TRG. At last, for cancer sequencing data, analysis showed that the TP53 was the most common mutation gene in the included studies. Recently, some studies demonstrated that the positive ctDNA was associated with the poor survival in pancreatic cancer [51], breast cancer [52], lung cancer and biliary tract cancer [53, 54]. Both our meta-analysis and Swathikan Chidambaram et al.’s meta-analysis [19] revealed the close association between the ctDNA and the prognosis of EC patients. However, the main aim of the previous meta-analysis was to assess sensitivity and specificity for diagnostic studies and surveillance purposes.

Development of new technologies such as ddPCR or NGS had greatly improved the sensitivity, specificity and precision for the detection of the ctDNA. The EC samples detected by ctDNA were assessed with those detected by tumor tissue in three studies [12, 34, 55]; and there was reasonable concordance between the two sets of results, among 86.3–66%. With the advent of the NGS technology, the DNA sequencing becomes dramatically easier and faster, and the NGS could also see when novel mutations happened during or after treatment [40]. The Illumina sequencers and Ion Torrent are two major NGS platforms; and the pace of this change is rapid with three new sequencing platforms having been released in recent years: CAPP-seq, Geneseeq and Signatera MRD. However, the sensitivity of the NGS is limited by the release of ctDNA into the plasma. In contrast to NGS, the ddPCR is likely to increase the detection rate but require personalized assays [44]. Combination of ddPCR and NGS showed encouraging result in localized colon cancer [56]. But there were no similar researches in EC. In addition, Azad et al. reported that the post-CRT ctDNA detection enables earlier identification of recurrence compared to the positron emission computed tomography (PET) [31]. The study of Cabalag et al. also showed ctDNA could provide additional prognostication over conventional staging investigation such as computed tomography (CT) and PET [42].

Liquid biopsy for obtaining ctDNA can provide information on neoadjuvant chemoradiotherapy (nCRT) pathological response and therefore might be a promising guide for these treatments. The nCRT is favored approach with evidence for improved pathologic complete response (pCR) rates and improved OS compared with surgery alone [57]. Meanwhile, some patients developed disease progression in the early stage of nCRT. The initial assessment by conventional imaging was unable to identify which patients will achieve durable clinical benefit. To guide appropriate treatment strategy, an accurate tumor monitoring modality that reflects tumor burden during neoadjuvant treatment is required for EC patients. In the CROSS trial, 30% of patients presented with distant metastases within two years after nCRT and surgery [7]. Our study showed the ctDNA little collected before surgery was associated with the pathological response, when we applied big panel (more 50 genes). In the trail of Morimoto et al., three out of four pathological responders became ctDNA negative after NAC, the ctDNA positive rate after NAC significantly correlated with the pathological response [41]; and Yang et al. demonstrated that ctDNA monitor might help identify which ESCC patients respond to chemoimmunotherapy [43]. Our study showed that the estimated MPR rates of patients changed positive to negative and kept negative from beginning to end were 50% and 51%, respectively, which were higher than the average MPR rates [58]. Therefore, Longitudinal ctDNA data for disease monitoring might have more clinical value.

The NGS-based multigene panel testing enables assessment of the mutational status of a few hundred genes associated with cancer pathogenesis. The Panel size depends not only on the number of genes tested, but also on the size of the region covered by the genes, so there are what we call large panel or small panel. Our meta-analysis showed that larger panel showed slight advantage in neoadjuvant therapy. At last, different gene mutation showed difference in the prognosis. PIK3CA mutation corresponded with shorter survival of 3.8 versus 13.6 months; while, BRAF genomic alterations corresponded with a median OS of 5.6 months versus 13.7 months in BRAF wildtype patients. Positive HER2 and EGFR were without prognostic value [12]. In the ESCC patients, the driver gene molecular mutation burden (MMB) yielded an area under the receiver operation characteristic (ROC) curve of 0.89 for predicting the response to nCT; and, the cfDNA copy number variations (CNVs) yielded an area under ROC curve of 1.0 for predicting the response to nCT [39].

### Limitations

There are a number of limitations to this meta-analysis. The main limitation is the heterogeneity of the included studies, which is reflected in the wide CIs. A random-effects model was adopted in an attempt to account for significant interstudy heterogeneity. Another drawback is that the sample collection times were different in the included studies, posing potential publication and selection bias. And for the results to be reliable, it is of fundamental importance that an international standardization be validated. Third, almost all studies included this meta-analysis are single-arm trials, and the findings are hypothesis-generating. Lack of large head-to-head randomized controlled trials prevents us from making a firm conclusion.

Although studies have suggested the ctDNA might serve as a prognostic and predictive biomarker for neoadjuvant therapy, there were few studies concentrating on the association between ctDNA and EC patients received neoadjuvant therapy.

## Conclusion

In conclusion, this meta-analysis suggested that ctDNA detection was associated with decreased OS and PFS in patients with esophageal cancer. A standardized technique needs to be established in order to introduce ctDNA analysis into routine clinical practice. A large number of clinical data are in favor of design more suitable panel for ctDNA detection. Longitudinal ctDNA monitoring might be a better strategy in the neoadjuvant therapy. In an era of personalized medicine, ctDNA could be a promising tool to individualize treatment planning and to improve outcomes in esophageal cancer.

### Electronic supplementary material

Below is the link to the electronic supplementary material.

Supplementary Material 1

Supplementary Material 2

## Data Availability

All data generated or analyzed during this study are included in this published article and its supplementary information files.

## Abbreviations

CI: Confidence Interval
HR: Hazard Ratio
OS: Overall survival
PFS: Progression free survival
PRISMA: Preferred Reporting Items for Systematic Reviews and Meta-Analyses statement
NOS: Modified Newcastle-Ottawa Scale
EC: Esophageal cancer
ctDNA: Circulating tumor DNA
NGS: Next-generation sequencing
ddPCR: Droplet digital polymerase chain reaction
PROSPERO: International Prospective Register of Systematic Reviews
