# Supplementary material for: An update of clinical value of circulating tumor DNA in esophageal cancer: a systematic review and meta-analysis
Source: BMC Cancer. 2024 Jan 24;24:129. doi: 10.1186/s12885-024-11879-6 (PMC10809487; doi:10.1186/s12885-024-11879-6)
Supplement: Supplementary file 2 — Supplementary Material 2 [file 12885_2024_11879_MOESM2_ESM.pdf]

The assessment of the risk of bias in included studies using the Newcastle–Ottawa scale

Study	Year	Selection (0–4)				Comparability (0–2)		Outcome (0–3)			Total
		REC	SNEC	AE	DO	SC	AF	AO	FU	AFU	
		1	1	0	1	1	1	1	1	0	7
Ococks E [27]	2021	1	1	0	1	1	1	1	1	1	8
Jia R [28]	2021	1	1	0	1	1	1	1	1	1	**8**
Azad TD [29]	2020	1	1	0	1	1	1	1	1	0	7
Davidson M [30]	2019	1	1	0	0	1	1	1	1	0	**6**
Openshaw MR [31]	2020	1	1	0	1	1	1	1	1	1	8
Maron SB [12]	2019	1	1	0	1	1	1	1	1	0	7
Luo H [32]	2016	1	1	0	1	0	1	1	1	0	**6**
Liu T [33]	2021	1	1	1	1	1	1	1	1	1	9
Hsieh CC [34]	2016	1	1	0	1	1	0	1	1	0	**6**
van Velzen MJM[14]	2022	1	0	0	1	1	1	1	1	0	**6**
Hofste LSM[35]	2022	0	1	1	1	1	1	1	1	0	7
Wang X[36]	2022	1	1	0	1	1	1	0	1	0	6
Zhang R[37]	2020	1	1	0	1	1	1	1	1	1	8
Eyck BM[38]	2022	1	1	1	1	0	1	1	1	1	8
Morimoto Y[39]	2023	0	1	0	1	0	1	1	1	1	6
Cabalag CS[40]	2022	0	1	1	0	0	1	1	1	1	6
Yang D[41]	2022	1	1	0	1	1	1	1	1	1	**8**
Wallander K[42]	2023	1	1	0	1	1	1	1	1	0	7
Fujisawa R[44]	2021	1	1	0	0	1	1	1	1	0	**6**
Mehta R[46]	2023	1	1	0	1	1	1	1	1	1	8
Lander EM[45]	2023	1	1	1	1	1	1	1	1	1	9
van den Ende T[43]	2023	1	1	0	1	1	0	1	1	0	**6**
Ococks E [27]	2021	1	0	0	1	1	1	1	1	0	**6**
Jia R [28]	2021	0	1	1	1	1	1	1	1	0	7
Azad TD [29]	2020	1	1	0	1	1	1	0	1	0	6
Davidson M [30]	2019	1	1	0	1	1	1	0	0	1	6
Openshaw MR [31]	2020	1	1	0	1	1	1	1	1	0	7

REC: Representativeness of the exposed cohort; SNEC: Selection of the nonexposed cohort; AE: Ascertainment of exposure; DO: Demonstration that outcome of interest was not present at start of study; SC: Study controls for age, sex; AF: Study controls for any additional factors (chemoradiotherapy, curative resection); AO: Assessment of outcome; FU: Follow-up long enough (36 M) for outcomes to occur; AFU: Adequacy of follow-up of cohorts (⩾90%). "1" means that the study is satisfied the item and "0" means the opposite situation
